# 2-[4-(Carbazol-9-yl)phen­yl]-1,3-diethyl-1,3-di­phenyl­guanidine

**DOI:** 10.1107/S1600536813014517

**Published:** 2013-06-08

**Authors:** Ioannis Tiritiris, Willi Kantlehner

**Affiliations:** a Institut für Organische Chemie, Universität Stuttgart, Pfaffenwaldring 55, 70569 Stuttgart, Germany; b Fakultät Chemie/Organische Chemie, Hochschule Aalen, Beethovenstrasse 1, D-73430 Aalen, Germany

## Abstract

In the title compound, C_35_H_32_N_4_, the C—N bond lengths in the guanidine part are 1.286 (3), 1.387 (2) and 1.414 (2) Å, indicating double- and single-bond character. The N—C—N angles are 114.48 (17), 118.78 (17) and 126.72 (17)°, showing a deviation of the CN_3_ plane from an ideal trigonal–planar geometry. The carbazole ring system is almost planar (r.m.s. deviation = 0.002 Å). In the crystal, mol­ecules are connected by weak C—H⋯N hydrogen bonds, generating a zigzag chain along the *ac* plane. Weak π–π inter­actions [centroid–centroid distance = 3.785 (1) Å] between two phenyl rings of the guanidine moiety are also present.

## Related literature

For synthesis and characterization of carbazole-based compounds for blue OLEDs, see: Agarwal *et al.* (2011 ▶). For the crystal structure of 9-(4-nitro­phen­yl)-9*H*-carbazole, see: Chen *et al.* (2005 ▶). For the crystal structure of carbazole, see: Gerkin & Reppart (1986 ▶). For synthesis and characterization of light-emitting carbazole derivatives, see: Thomas *et al.* (2001 ▶). For the crystal structure of *N*,*N*,*N*′,*N*′-tetra­methyl-*N*′′-[2-(*N*′,*N*′,*N*′′,*N*′′-tetra­methyl­guanidino)eth­yl]guanidine, see: Tiritiris & Kantlehner (2012 ▶).
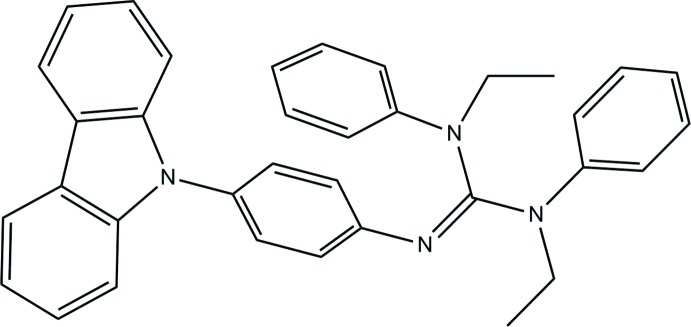


## Experimental

### 

#### Crystal data

C_35_H_32_N_4_
*M* *_r_* = 508.65
Orthorhombic, 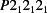

*a* = 9.4741 (2) Å
*b* = 15.9500 (6) Å
*c* = 17.9102 (8) Å
*V* = 2706.45 (17) Å^3^
*Z* = 4
Mo *K*α radiation
μ = 0.07 mm^−1^
*T* = 100 K
0.19 × 0.15 × 0.12 mm

#### Data collection

Bruker-Nonius KappaCCD diffractometer
6390 measured reflections
3588 independent reflections
3109 reflections with *I* > 2σ(*I*)
*R*_int_ = 0.032

#### Refinement

*R*[*F* ^2^ > 2σ(*F* ^2^)] = 0.038
*wR*(*F* ^2^) = 0.086
*S* = 1.04
3588 reflections
354 parameters
H-atom parameters constrained
Δρ_max_ = 0.18 e Å^−3^
Δρ_min_ = −0.20 e Å^−3^

### 

Data collection: *COLLECT* (Hooft, 2004 ▶); cell refinement: *SCALEPACK* (Otwinowski & Minor, 1997 ▶); data reduction: *SCALEPACK*; program(s) used to solve structure: *SHELXS97* (Sheldrick, 2008 ▶); program(s) used to refine structure: *SHELXL97* (Sheldrick, 2008 ▶); molecular graphics: *DIAMOND* (Brandenburg & Putz, 2005 ▶); software used to prepare material for publication: *SHELXL97*.

## Supplementary Material

Crystal structure: contains datablock(s) I, global. DOI: 10.1107/S1600536813014517/zl2551sup1.cif

Structure factors: contains datablock(s) I. DOI: 10.1107/S1600536813014517/zl2551Isup2.hkl

Supplementary material file. DOI: 10.1107/S1600536813014517/zl2551Isup3.cml

Additional supplementary materials:  crystallographic information; 3D view; checkCIF report

## Figures and Tables

**Table 1:** Hydrogen-bond geometry (Å, °)

*D*—H⋯*A*	*D*—H	H⋯*A*	*D*⋯*A*	*D*—H⋯*A*
C28—H28*A*⋯N3^i^	0.95	2.79	3.593 (3)	143

Symmetry code: (i) 
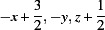
.

**Fig. 1.**
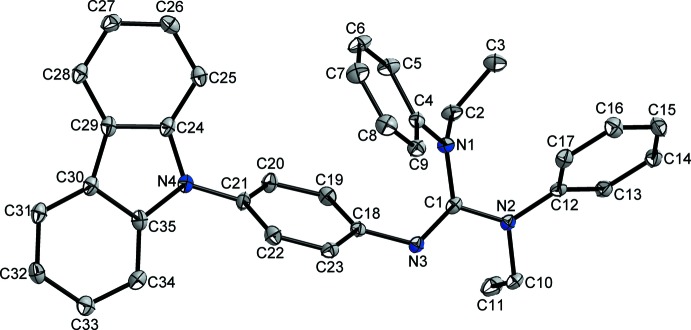
The structure of the title compound with atom labels and 50% probability displacement ellipsoids. All hydrogen atoms were omitted for clarity.

**Fig. 2.**
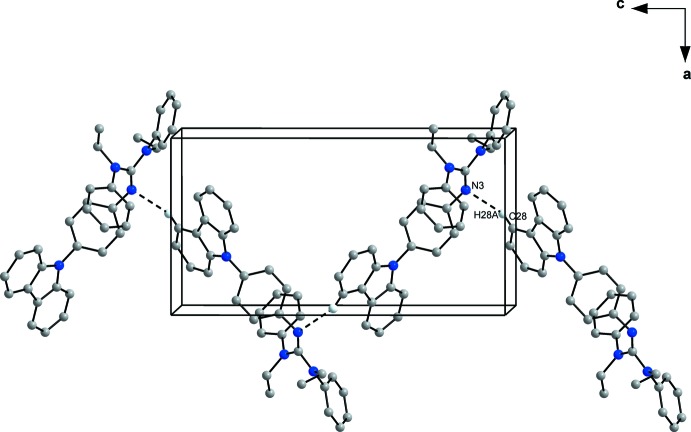
C–H···N hydrogen bonds between the guanidine molecules, ac-view. The hydrogen bonds are indicated by dashed lines.

## supplementary crystallographic information

### Comment

Carbazole derivatives play an important role as materials for small-molecule or polymer organic light-emitting diodes (OLEDs). They are known for their intense blue luminescence (Thomas *et al.*, 2001) and several types of carbazole-based compounds are already used in OLEDs (Agarwal *et al.*, 2011). The disadvantage of blue emitting materials in blue OLEDs is their lower efficiency compared to green or red OLED materials, due to their limited stability, shorter lifetime and lower color purity. In search of new more stable blue emitting materials, we synthesized a new type of carbazole derivative by combination of an aryl substituted carbazole with a guanidine moiety, the crystal structure of which is presented here.

According to the structure analysis, the C1–N3 bond in the guanidine unit is 1.286 (3) Å, indicating double bond character. The bond lengths C1–N2 = 1.387 (2) Å and C1–N1 = 1.414 (2) Å are elongated and characteristic for C–N imine single bonds. The N–C1–N angles are 114.48 (17)° (N1–C1–N2), 118.78 (17)° (N2–C1–N3) and 126.72 (17)° (N1–C1–N3), showing a deviation of the CN_3_ plane from an ideal trigonal planar geometry (Fig. 1). Similar bond lengths and angles of the guanidine CN_3_ part have been found by structure analysis for *N*,*N*,*N'*,*N'*-tetramethyl-*N''*-[2-(*N'*,*N'*,*N''*,*N''*-tetramethylguanidino)-ethyl]-guanidine (Tiritiris & Kantlehner, 2012). The carbazole ring system is planar and the bond lengths and angles are in good agreement with the data from the X-ray analysis of unsubstituted carbazole (Gerkin & Reppart, 1986). The dihedral angle between the planes C35/N4/C24 and C22/C21/C20 is 57.9 (2)°, indicating an only small deviation from the value found for 9-(4-nitrophenyl)-9*H*-carbazole [dihedral angle between the C/N/C and C/C/C plane = 53.08 (4)°] (Chen *et al.*, 2005). Weak C–H···N hydrogen bonds are found between aromatic hydrogen atoms of the carbazole moiety and the free nitrogen atoms of neighboring guanidine molecules [*d*(H···N) = 2.79 Å] (Tab. 1), generating a zig zag chain along the *ac*-plane (Fig. 2). Finally, weak π–π interactions between two phenyl rings of the guanidine unit [*Cg*1 = C4–C9; *Cg*2 = C18–C23; *d*(*Cg*1···*Cg*2) = 3.785 Å] are also present.

### Experimental

One equivalent of *N*,*N'*-diethyl-*N*,*N'*-diphenylchloro formamidinium-chloride (synthesized from *N*,*N'*-diethyl-*N*,*N'*-diphenylthiourea and phosgene) was reacted with one equivalent of 9-(4-aminophenyl)-9*H*-carbazole (Alfa Aesar) in acetonitrile, in the presence of one equivalent triethylamine, at 273 K. The obtained mixture consisting of the guanidinium chloride and triethylammonium chloride was reacted in the next step with an excess of an aqueous sodium hydroxide solution at 273 K. After extraction of the guanidine with diethyl ether from the water phase, the solvent was evaporated and the title compound was isolated in form of a colourless solid. Single crystals have been obtained by recrystallization from a saturated acetonitrile solution.

### Refinement

The title compound crystallizes in the non-centrosymmetric space group *P2_1_2_1_2_1_*; however, in the absence of significant anomalous scattering effects, the Flack parameter is essentially meaningless. Accordingly, Friedel pairs were merged. The hydrogen atoms of the methyl groups were allowed to rotate with a fixed angle around the C–N bond to best fit the experimental electron density, with *U*_iso_(H) set to 1.5 *U*_eq_(C) and d(C—H) = 0.98 Å. The remaining H atoms were placed in calculated positions with d(C—H) = 0.99 Å (H atoms in CH_2_ groups) and (C—H) = 0.95 Å (H atoms in aromatic rings). They were included in the refinement in the riding model approximation, with *U*_iso_(H) set to 1.2 *U*_eq_(C).

### Crystal data

**Table:**

C_35_H_32_N_4_	*F*(000) = 1080
*M**_r_* = 508.65	*D*_x_ = 1.248 Mg m^−^^3^
Orthorhombic, *P*2_1_2_1_2_1_	Mo *K*α radiation, λ = 0.71073 Å
Hall symbol: P 2ac 2ab	Cell parameters from 33481 reflections
*a* = 9.4741 (2) Å	θ = 0.4–27.9°
*b* = 15.9500 (6) Å	µ = 0.07 mm^−^^1^
*c* = 17.9102 (8) Å	*T* = 100 K
*V* = 2706.45 (17) Å^3^	Block, colorless
*Z* = 4	0.19 × 0.15 × 0.12 mm

### Data collection

**Table:**

Bruker-Nonius KappaCCD diffractometer	3109 reflections with *I* > 2σ(*I*)
Radiation source: sealed tube	*R*_int_ = 0.032
Graphite monochromator	θ_max_ = 27.9°, θ_min_ = 2.8°
φ scans, and ω scans	*h* = −12→12
6390 measured reflections	*k* = −20→20
3588 independent reflections	*l* = −23→23

### Refinement

**Table:**

Refinement on *F*^2^	Primary atom site location: structure-invariant direct methods
Least-squares matrix: full	Secondary atom site location: difference Fourier map
*R*[*F*^2^ > 2σ(*F*^2^)] = 0.038	Hydrogen site location: difference Fourier map
*wR*(*F*^2^) = 0.086	H-atom parameters constrained
*S* = 1.04	*w* = 1/[σ^2^(*F*_o_^2^) + (0.0341*P*)^2^ + 0.8137*P*] where *P* = (*F*_o_^2^ + 2*F*_c_^2^)/3
3588 reflections	(Δ/σ)_max_ < 0.001
354 parameters	Δρ_max_ = 0.18 e Å^−^^3^
0 restraints	Δρ_min_ = −0.20 e Å^−^^3^

### Special details

**Table:**

Geometry. All e.s.d.'s (except the e.s.d. in the dihedral angle between two l.s. planes) are estimated using the full covariance matrix. The cell e.s.d.'s are taken into account individually in the estimation of e.s.d.'s in distances, angles and torsion angles; correlations between e.s.d.'s in cell parameters are only used when they are defined by crystal symmetry. An approximate (isotropic) treatment of cell e.s.d.'s is used for estimating e.s.d.'s involving l.s. planes.
Refinement. Refinement of *F*^2^ against ALL reflections. The weighted *R*-factor *wR* and goodness of fit *S* are based on *F*^2^, conventional *R*-factors *R* are based on *F*, with *F* set to zero for negative *F*^2^. The threshold expression of *F*^2^ > σ(*F*^2^) is used only for calculating *R*-factors(gt) *etc*. and is not relevant to the choice of reflections for refinement. *R*-factors based on *F*^2^ are statistically about twice as large as those based on *F*, and *R*- factors based on ALL data will be even larger.

### Fractional atomic coordinates and isotropic or equivalent isotropic displacement parameters (Å^2^)

**Table:**

	*x*	*y*	*z*	*U*_iso_*/*U*_eq_	
C1	0.2398 (2)	0.01022 (12)	0.15167 (10)	0.0136 (4)	
N1	0.21769 (17)	0.08232 (11)	0.19605 (9)	0.0142 (3)	
C2	0.0991 (2)	0.07985 (13)	0.24863 (11)	0.0184 (4)	
H2A	0.1373	0.0796	0.3001	0.022*	
H2B	0.0474	0.0265	0.2413	0.022*	
C3	−0.0054 (2)	0.15231 (15)	0.24149 (13)	0.0234 (5)	
H3A	0.0455	0.2057	0.2445	0.035*	
H3B	−0.0747	0.1492	0.2820	0.035*	
H3C	−0.0542	0.1486	0.1933	0.035*	
C4	0.3269 (2)	0.14186 (13)	0.20511 (11)	0.0150 (4)	
C5	0.3325 (2)	0.19439 (14)	0.26785 (12)	0.0217 (5)	
H5A	0.2611	0.1910	0.3050	0.026*	
C6	0.4426 (3)	0.25155 (14)	0.27582 (13)	0.0253 (5)	
H6A	0.4451	0.2872	0.3183	0.030*	
C7	0.5483 (2)	0.25736 (14)	0.22300 (13)	0.0249 (5)	
H7A	0.6245	0.2953	0.2297	0.030*	
C8	0.5414 (2)	0.20701 (14)	0.16015 (12)	0.0213 (4)	
H8A	0.6130	0.2111	0.1232	0.026*	
C9	0.4312 (2)	0.15069 (13)	0.15042 (11)	0.0180 (4)	
H9A	0.4267	0.1178	0.1062	0.022*	
N2	0.12723 (17)	−0.01060 (11)	0.10577 (9)	0.0155 (3)	
C10	0.1179 (2)	−0.09757 (13)	0.07804 (11)	0.0169 (4)	
H10A	0.2127	−0.1161	0.0616	0.020*	
H10B	0.0545	−0.0991	0.0341	0.020*	
C11	0.0633 (3)	−0.15786 (14)	0.13647 (13)	0.0258 (5)	
H11A	0.1243	−0.1556	0.1806	0.039*	
H11B	0.0634	−0.2149	0.1162	0.039*	
H11C	−0.0331	−0.1421	0.1505	0.039*	
C12	0.0369 (2)	0.05132 (13)	0.07409 (11)	0.0153 (4)	
C13	−0.1054 (2)	0.03251 (14)	0.06186 (11)	0.0173 (4)	
H13A	−0.1416	−0.0209	0.0755	0.021*	
C14	−0.1937 (2)	0.09201 (14)	0.02976 (12)	0.0204 (4)	
H14A	−0.2902	0.0789	0.0212	0.024*	
C15	−0.1423 (2)	0.17052 (14)	0.01001 (12)	0.0224 (5)	
H15A	−0.2031	0.2110	−0.0118	0.027*	
C16	−0.0017 (2)	0.18903 (14)	0.02245 (12)	0.0203 (4)	
H16A	0.0338	0.2428	0.0094	0.024*	
C17	0.0877 (2)	0.13042 (13)	0.05360 (11)	0.0171 (4)	
H17A	0.1843	0.1438	0.0612	0.020*	
N3	0.34992 (17)	−0.03696 (11)	0.15142 (9)	0.0156 (3)	
C18	0.4536 (2)	−0.02744 (12)	0.20755 (11)	0.0151 (4)	
C19	0.4217 (2)	−0.02753 (13)	0.28357 (11)	0.0167 (4)	
H19A	0.3265	−0.0337	0.2994	0.020*	
C20	0.5281 (2)	−0.01872 (13)	0.33636 (11)	0.0179 (4)	
H20A	0.5060	−0.0200	0.3881	0.021*	
C21	0.6666 (2)	−0.00799 (13)	0.31334 (11)	0.0167 (4)	
C22	0.7001 (2)	−0.01022 (14)	0.23777 (12)	0.0182 (4)	
H22A	0.7953	−0.0039	0.2221	0.022*	
C23	0.5944 (2)	−0.02163 (13)	0.18542 (11)	0.0166 (4)	
H23A	0.6180	−0.0255	0.1340	0.020*	
N4	0.77493 (17)	0.00757 (11)	0.36707 (9)	0.0176 (4)	
C24	0.7771 (2)	0.07533 (13)	0.41611 (11)	0.0157 (4)	
C25	0.6772 (2)	0.13854 (13)	0.42557 (12)	0.0186 (4)	
H25A	0.5941	0.1408	0.3959	0.022*	
C26	0.7044 (2)	0.19800 (14)	0.48025 (12)	0.0209 (4)	
H26A	0.6380	0.2417	0.4884	0.025*	
C27	0.8272 (2)	0.19524 (14)	0.52382 (12)	0.0214 (4)	
H27A	0.8420	0.2367	0.5611	0.026*	
C28	0.9275 (2)	0.13299 (13)	0.51332 (11)	0.0178 (4)	
H28A	1.0107	0.1314	0.5429	0.021*	
C29	0.9032 (2)	0.07256 (13)	0.45807 (11)	0.0156 (4)	
C30	0.9823 (2)	0.00075 (13)	0.43164 (10)	0.0160 (4)	
C31	1.1140 (2)	−0.03379 (14)	0.44977 (11)	0.0184 (4)	
H31A	1.1717	−0.0087	0.4870	0.022*	
C32	1.1585 (2)	−0.10510 (14)	0.41248 (11)	0.0199 (4)	
H32A	1.2481	−0.1286	0.4238	0.024*	
C33	1.0730 (2)	−0.14313 (14)	0.35808 (12)	0.0207 (4)	
H33A	1.1058	−0.1922	0.3335	0.025*	
C34	0.9419 (2)	−0.11072 (14)	0.33932 (12)	0.0193 (4)	
H34A	0.8836	−0.1371	0.3031	0.023*	
C35	0.8993 (2)	−0.03802 (13)	0.37572 (11)	0.0163 (4)	

### Atomic displacement parameters (Å^2^)

**Table:**

	*U*^11^	*U*^22^	*U*^33^	*U*^12^	*U*^13^	*U*^23^
C1	0.0166 (9)	0.0133 (9)	0.0109 (9)	−0.0038 (8)	−0.0003 (7)	0.0004 (7)
N1	0.0142 (7)	0.0138 (8)	0.0145 (8)	−0.0010 (7)	0.0013 (6)	−0.0017 (7)
C2	0.0200 (10)	0.0172 (10)	0.0181 (10)	−0.0022 (9)	0.0046 (8)	−0.0023 (8)
C3	0.0228 (10)	0.0241 (11)	0.0232 (11)	0.0019 (10)	0.0055 (9)	−0.0027 (9)
C4	0.0166 (9)	0.0119 (9)	0.0164 (9)	−0.0013 (8)	−0.0028 (8)	0.0014 (8)
C5	0.0262 (11)	0.0202 (11)	0.0187 (10)	−0.0018 (9)	0.0038 (9)	−0.0031 (9)
C6	0.0351 (12)	0.0180 (11)	0.0228 (11)	−0.0051 (10)	−0.0002 (10)	−0.0074 (9)
C7	0.0257 (11)	0.0172 (11)	0.0318 (12)	−0.0080 (9)	−0.0016 (10)	0.0001 (9)
C8	0.0211 (10)	0.0191 (11)	0.0237 (11)	−0.0038 (9)	0.0030 (9)	0.0031 (9)
C9	0.0215 (10)	0.0154 (10)	0.0170 (9)	−0.0017 (8)	−0.0009 (8)	0.0008 (8)
N2	0.0152 (8)	0.0144 (8)	0.0169 (8)	−0.0002 (7)	−0.0041 (7)	−0.0019 (7)
C10	0.0183 (10)	0.0159 (10)	0.0166 (9)	−0.0014 (8)	−0.0028 (8)	−0.0044 (8)
C11	0.0358 (13)	0.0187 (11)	0.0228 (11)	−0.0047 (10)	−0.0012 (10)	−0.0020 (9)
C12	0.0163 (9)	0.0175 (10)	0.0120 (9)	0.0016 (8)	0.0008 (8)	−0.0031 (8)
C13	0.0168 (10)	0.0184 (10)	0.0167 (9)	−0.0014 (8)	−0.0008 (8)	−0.0023 (8)
C14	0.0155 (10)	0.0263 (11)	0.0193 (10)	0.0013 (8)	−0.0024 (8)	−0.0049 (9)
C15	0.0223 (10)	0.0233 (11)	0.0215 (10)	0.0077 (9)	−0.0024 (9)	0.0026 (9)
C16	0.0224 (10)	0.0201 (11)	0.0185 (10)	0.0001 (9)	0.0015 (8)	0.0037 (9)
C17	0.0146 (9)	0.0228 (11)	0.0138 (9)	−0.0021 (8)	0.0013 (8)	0.0002 (8)
N3	0.0143 (8)	0.0153 (8)	0.0172 (8)	−0.0003 (7)	−0.0031 (7)	−0.0016 (7)
C18	0.0159 (9)	0.0112 (9)	0.0182 (10)	0.0008 (8)	−0.0043 (8)	−0.0005 (8)
C19	0.0139 (9)	0.0170 (10)	0.0193 (9)	−0.0014 (8)	0.0017 (8)	0.0015 (8)
C20	0.0193 (10)	0.0203 (11)	0.0141 (9)	−0.0005 (8)	−0.0004 (8)	0.0023 (8)
C21	0.0184 (10)	0.0169 (10)	0.0149 (9)	−0.0010 (8)	−0.0057 (8)	0.0008 (8)
C22	0.0138 (9)	0.0203 (11)	0.0204 (10)	−0.0011 (8)	0.0013 (8)	0.0004 (9)
C23	0.0189 (10)	0.0170 (10)	0.0140 (9)	−0.0001 (8)	0.0007 (8)	−0.0017 (8)
N4	0.0163 (8)	0.0202 (9)	0.0163 (8)	0.0012 (7)	−0.0035 (7)	−0.0017 (7)
C24	0.0175 (9)	0.0178 (10)	0.0118 (9)	−0.0032 (8)	0.0001 (8)	0.0026 (8)
C25	0.0178 (10)	0.0194 (10)	0.0186 (10)	−0.0001 (8)	−0.0008 (8)	0.0037 (8)
C26	0.0216 (10)	0.0175 (10)	0.0236 (11)	0.0033 (9)	0.0002 (9)	0.0023 (9)
C27	0.0284 (11)	0.0160 (10)	0.0197 (10)	−0.0023 (9)	−0.0013 (9)	−0.0020 (9)
C28	0.0202 (10)	0.0188 (10)	0.0143 (9)	−0.0036 (8)	−0.0016 (8)	0.0023 (8)
C29	0.0171 (9)	0.0156 (10)	0.0141 (9)	−0.0033 (8)	−0.0011 (8)	0.0053 (8)
C30	0.0184 (9)	0.0167 (10)	0.0129 (9)	−0.0029 (8)	−0.0002 (8)	0.0029 (8)
C31	0.0192 (10)	0.0207 (10)	0.0154 (9)	−0.0033 (9)	−0.0020 (8)	0.0031 (8)
C32	0.0177 (10)	0.0233 (11)	0.0186 (10)	0.0016 (9)	−0.0016 (8)	0.0051 (9)
C33	0.0232 (10)	0.0208 (10)	0.0180 (10)	0.0014 (9)	0.0013 (8)	0.0022 (9)
C34	0.0210 (10)	0.0210 (10)	0.0158 (9)	−0.0023 (9)	−0.0017 (8)	−0.0017 (8)
C35	0.0160 (9)	0.0182 (10)	0.0148 (9)	−0.0015 (8)	0.0003 (8)	0.0048 (8)

### Geometric parameters (Å, º)

**Table:**

C1—N3	1.286 (3)	C16—C17	1.379 (3)
C1—N2	1.387 (2)	C16—H16A	0.9500
C1—N1	1.414 (2)	C17—H17A	0.9500
N1—C4	1.413 (2)	N3—C18	1.414 (2)
N1—C2	1.466 (2)	C18—C23	1.395 (3)
C2—C3	1.527 (3)	C18—C19	1.395 (3)
C2—H2A	0.9900	C19—C20	1.390 (3)
C2—H2B	0.9900	C19—H19A	0.9500
C3—H3A	0.9800	C20—C21	1.386 (3)
C3—H3B	0.9800	C20—H20A	0.9500
C3—H3C	0.9800	C21—C22	1.391 (3)
C4—C9	1.399 (3)	C21—N4	1.429 (3)
C4—C5	1.403 (3)	C22—C23	1.383 (3)
C5—C6	1.393 (3)	C22—H22A	0.9500
C5—H5A	0.9500	C23—H23A	0.9500
C6—C7	1.380 (3)	N4—C24	1.393 (3)
C6—H6A	0.9500	N4—C35	1.393 (3)
C7—C8	1.384 (3)	C24—C25	1.393 (3)
C7—H7A	0.9500	C24—C29	1.412 (3)
C8—C9	1.388 (3)	C25—C26	1.388 (3)
C8—H8A	0.9500	C25—H25A	0.9500
C9—H9A	0.9500	C26—C27	1.402 (3)
N2—C12	1.425 (3)	C26—H26A	0.9500
N2—C10	1.476 (3)	C27—C28	1.387 (3)
C10—C11	1.512 (3)	C27—H27A	0.9500
C10—H10A	0.9900	C28—C29	1.400 (3)
C10—H10B	0.9900	C28—H28A	0.9500
C11—H11A	0.9800	C29—C30	1.448 (3)
C11—H11B	0.9800	C30—C31	1.402 (3)
C11—H11C	0.9800	C30—C35	1.416 (3)
C12—C13	1.398 (3)	C31—C32	1.385 (3)
C12—C17	1.399 (3)	C31—H31A	0.9500
C13—C14	1.390 (3)	C32—C33	1.405 (3)
C13—H13A	0.9500	C32—H32A	0.9500
C14—C15	1.389 (3)	C33—C34	1.387 (3)
C14—H14A	0.9500	C33—H33A	0.9500
C15—C16	1.382 (3)	C34—C35	1.390 (3)
C15—H15A	0.9500	C34—H34A	0.9500
			
N3—C1—N2	118.78 (17)	C17—C16—H16A	119.6
N3—C1—N1	126.72 (17)	C15—C16—H16A	119.6
N2—C1—N1	114.48 (17)	C16—C17—C12	120.40 (19)
C4—N1—C1	120.18 (16)	C16—C17—H17A	119.8
C4—N1—C2	120.33 (16)	C12—C17—H17A	119.8
C1—N1—C2	116.92 (16)	C1—N3—C18	119.89 (17)
N1—C2—C3	115.00 (17)	C23—C18—C19	119.02 (18)
N1—C2—H2A	108.5	C23—C18—N3	118.02 (17)
C3—C2—H2A	108.5	C19—C18—N3	122.89 (18)
N1—C2—H2B	108.5	C20—C19—C18	120.42 (18)
C3—C2—H2B	108.5	C20—C19—H19A	119.8
H2A—C2—H2B	107.5	C18—C19—H19A	119.8
C2—C3—H3A	109.5	C21—C20—C19	119.81 (18)
C2—C3—H3B	109.5	C21—C20—H20A	120.1
H3A—C3—H3B	109.5	C19—C20—H20A	120.1
C2—C3—H3C	109.5	C20—C21—C22	120.16 (18)
H3A—C3—H3C	109.5	C20—C21—N4	120.08 (18)
H3B—C3—H3C	109.5	C22—C21—N4	119.74 (18)
C9—C4—C5	118.28 (19)	C23—C22—C21	119.88 (19)
C9—C4—N1	120.29 (18)	C23—C22—H22A	120.1
C5—C4—N1	121.42 (18)	C21—C22—H22A	120.1
C6—C5—C4	120.1 (2)	C22—C23—C18	120.53 (18)
C6—C5—H5A	119.9	C22—C23—H23A	119.7
C4—C5—H5A	119.9	C18—C23—H23A	119.7
C7—C6—C5	121.1 (2)	C24—N4—C35	108.82 (16)
C7—C6—H6A	119.4	C24—N4—C21	124.74 (17)
C5—C6—H6A	119.4	C35—N4—C21	126.29 (17)
C6—C7—C8	119.0 (2)	N4—C24—C25	128.93 (18)
C6—C7—H7A	120.5	N4—C24—C29	108.87 (17)
C8—C7—H7A	120.5	C25—C24—C29	122.20 (19)
C7—C8—C9	120.9 (2)	C26—C25—C24	117.01 (19)
C7—C8—H8A	119.6	C26—C25—H25A	121.5
C9—C8—H8A	119.6	C24—C25—H25A	121.5
C8—C9—C4	120.58 (19)	C25—C26—C27	121.7 (2)
C8—C9—H9A	119.7	C25—C26—H26A	119.1
C4—C9—H9A	119.7	C27—C26—H26A	119.1
C1—N2—C12	122.14 (17)	C28—C27—C26	121.1 (2)
C1—N2—C10	118.07 (16)	C28—C27—H27A	119.5
C12—N2—C10	118.78 (15)	C26—C27—H27A	119.5
N2—C10—C11	112.65 (16)	C27—C28—C29	118.40 (19)
N2—C10—H10A	109.1	C27—C28—H28A	120.8
C11—C10—H10A	109.1	C29—C28—H28A	120.8
N2—C10—H10B	109.1	C28—C29—C24	119.58 (19)
C11—C10—H10B	109.1	C28—C29—C30	133.63 (19)
H10A—C10—H10B	107.8	C24—C29—C30	106.79 (17)
C10—C11—H11A	109.5	C31—C30—C35	119.12 (19)
C10—C11—H11B	109.5	C31—C30—C29	134.08 (19)
H11A—C11—H11B	109.5	C35—C30—C29	106.80 (17)
C10—C11—H11C	109.5	C32—C31—C30	118.81 (19)
H11A—C11—H11C	109.5	C32—C31—H31A	120.6
H11B—C11—H11C	109.5	C30—C31—H31A	120.6
C13—C12—C17	118.94 (19)	C31—C32—C33	120.9 (2)
C13—C12—N2	119.52 (19)	C31—C32—H32A	119.5
C17—C12—N2	121.52 (18)	C33—C32—H32A	119.5
C14—C13—C12	119.9 (2)	C34—C33—C32	121.6 (2)
C14—C13—H13A	120.0	C34—C33—H33A	119.2
C12—C13—H13A	120.0	C32—C33—H33A	119.2
C15—C14—C13	120.6 (2)	C33—C34—C35	117.2 (2)
C15—C14—H14A	119.7	C33—C34—H34A	121.4
C13—C14—H14A	119.7	C35—C34—H34A	121.4
C16—C15—C14	119.3 (2)	C34—C35—N4	128.95 (19)
C16—C15—H15A	120.3	C34—C35—C30	122.34 (19)
C14—C15—H15A	120.3	N4—C35—C30	108.70 (18)
C17—C16—C15	120.8 (2)		
			
N3—C1—N1—C4	43.7 (3)	C19—C20—C21—N4	175.17 (19)
N2—C1—N1—C4	−137.85 (19)	C20—C21—C22—C23	1.2 (3)
N3—C1—N1—C2	−118.2 (2)	N4—C21—C22—C23	−177.19 (19)
N2—C1—N1—C2	60.3 (2)	C21—C22—C23—C18	2.7 (3)
C4—N1—C2—C3	72.3 (2)	C19—C18—C23—C22	−4.5 (3)
C1—N1—C2—C3	−125.84 (19)	N3—C18—C23—C22	178.43 (19)
C1—N1—C4—C9	26.1 (3)	C20—C21—N4—C24	−59.2 (3)
C2—N1—C4—C9	−172.63 (18)	C22—C21—N4—C24	119.2 (2)
C1—N1—C4—C5	−155.02 (19)	C20—C21—N4—C35	125.6 (2)
C2—N1—C4—C5	6.2 (3)	C22—C21—N4—C35	−56.0 (3)
C9—C4—C5—C6	−2.1 (3)	C35—N4—C24—C25	178.3 (2)
N1—C4—C5—C6	179.0 (2)	C21—N4—C24—C25	2.4 (3)
C4—C5—C6—C7	−0.5 (3)	C35—N4—C24—C29	−1.2 (2)
C5—C6—C7—C8	2.0 (3)	C21—N4—C24—C29	−177.03 (18)
C6—C7—C8—C9	−0.8 (3)	N4—C24—C25—C26	178.5 (2)
C7—C8—C9—C4	−1.9 (3)	C29—C24—C25—C26	−2.1 (3)
C5—C4—C9—C8	3.3 (3)	C24—C25—C26—C27	0.5 (3)
N1—C4—C9—C8	−177.79 (18)	C25—C26—C27—C28	0.6 (3)
N3—C1—N2—C12	−148.86 (18)	C26—C27—C28—C29	−0.1 (3)
N1—C1—N2—C12	32.6 (3)	C27—C28—C29—C24	−1.5 (3)
N3—C1—N2—C10	19.5 (3)	C27—C28—C29—C30	179.5 (2)
N1—C1—N2—C10	−159.04 (16)	N4—C24—C29—C28	−177.84 (18)
C1—N2—C10—C11	77.5 (2)	C25—C24—C29—C28	2.7 (3)
C12—N2—C10—C11	−113.7 (2)	N4—C24—C29—C30	1.4 (2)
C1—N2—C12—C13	−147.79 (19)	C25—C24—C29—C30	−178.07 (18)
C10—N2—C12—C13	43.9 (3)	C28—C29—C30—C31	−2.2 (4)
C1—N2—C12—C17	33.3 (3)	C24—C29—C30—C31	178.7 (2)
C10—N2—C12—C17	−134.96 (19)	C28—C29—C30—C35	178.0 (2)
C17—C12—C13—C14	0.1 (3)	C24—C29—C30—C35	−1.2 (2)
N2—C12—C13—C14	−178.86 (18)	C35—C30—C31—C32	−0.3 (3)
C12—C13—C14—C15	−0.4 (3)	C29—C30—C31—C32	179.9 (2)
C13—C14—C15—C16	0.1 (3)	C30—C31—C32—C33	−0.8 (3)
C14—C15—C16—C17	0.5 (3)	C31—C32—C33—C34	0.4 (3)
C15—C16—C17—C12	−0.8 (3)	C32—C33—C34—C35	1.1 (3)
C13—C12—C17—C16	0.6 (3)	C33—C34—C35—N4	179.31 (19)
N2—C12—C17—C16	179.45 (18)	C33—C34—C35—C30	−2.2 (3)
N2—C1—N3—C18	−167.41 (17)	C24—N4—C35—C34	179.1 (2)
N1—C1—N3—C18	10.9 (3)	C21—N4—C35—C34	−5.1 (3)
C1—N3—C18—C23	−131.2 (2)	C24—N4—C35—C30	0.4 (2)
C1—N3—C18—C19	51.9 (3)	C21—N4—C35—C30	176.20 (18)
C23—C18—C19—C20	2.5 (3)	C31—C30—C35—C34	1.8 (3)
N3—C18—C19—C20	179.39 (19)	C29—C30—C35—C34	−178.30 (18)
C18—C19—C20—C21	1.4 (3)	C31—C30—C35—N4	−179.41 (17)
C19—C20—C21—C22	−3.2 (3)	C29—C30—C35—N4	0.5 (2)

### Hydrogen-bond geometry (Å, º)

**Table:**

*D*—H···*A*	*D*—H	H···*A*	*D*···*A*	*D*—H···*A*
C28—H28*A*···N3^i^	0.95	2.79	3.593 (3)	143

Symmetry code: (i) −*x*+3/2, −*y*, *z*+1/2.
